# Navigating COVID-19: Association between public perceptions of preventive measures and delayed or foregone care among young and middle-aged Korean adults in the early phase of the pandemic

**DOI:** 10.1371/journal.pone.0344209

**Published:** 2026-03-13

**Authors:** Jongnam Hwang

**Affiliations:** Division of Social Welfare & Health Administration, Wonkwang University, Iksan, Korea; Czech University of Life Sciences Prague: Ceska Zemedelska Univerzita v Praze, CZECHIA

## Abstract

The COVID-19 pandemic has had a profound impact on healthcare systems worldwide, leading to significant disruptions in healthcare services and an increase in delayed or foregone care. South Korea has been no exception. While the importance of public perceptions of COVID-19 and healthcare use has been recognized, how such perceptions shape healthcare-seeking behaviour remains underexplored in the Korean context. The aim of this study was to examine the association between public perceptions of preventive measures and instances of delayed or foregone care during the early phase of the pandemic in 2020. This study utilized data from the 2020 Koreans’ Happiness Survey (KHS), a nationally representative cross-sectional dataset. The analysis included a total of 11,307 respondents, aged 19–64, who completed both the general and COVID-19 modules of the survey. To assess delayed or foregone care, self-reported responses were used. Public perceptions of COVID-19 preventive measures were categorised into four groups based on reported trust and understanding. The association between public perceptions of the preventive measures and delayed or foregone care was assessed using logistic regression analyses, which were adjusted for sociodemographic, health status, and COVID-19-related characteristics. The results of this study revealed that Korean adults, who both understood and trusted COVID-19 preventive measures, had significantly lower odds of delayed or foregone care (OR: 0.49, 95% CI: 0.36–0.65) as compared with those who lacked both. Chronic conditions, worsened health status, and lower income were associated with a higher likelihood of delayed or foregone care. These findings highlight that both cognitive and affective dimensions of public perceptions may have shaped the use of healthcare services during the public health crisis. During future public health emergencies, strengthening public trust and risk communication may be important to mitigate barriers to timely access to healthcare.

## Introduction

Healthcare services worldwide have faced unprecedented challenges in the wake of the global COVID-19 pandemic [1]. Most countries have grappled with difficulties as hospitals prioritized treating the most critically ill individuals with COVID-19 [2,3]. Inadvertently, this necessary global focus on pandemic response led to significant backlogs of individuals in need of other essential healthcare services, resulting in delayed or foregone care with unexpected health consequences [4,5]. These challenges affected routine care services, such as child health visits, immunizations, and chronic disease management, as well as emergency and urgent care requiring immediate intervention [6]. While delayed or foregone care has been partially attributed to supply-side constraints, such as limited availability of services or staff shortages, growing evidence suggests that perceptions of the pandemic and related preventive measures were associated with individual decisions to seek or avoid healthcare services [7,8].

Korea, which operates a social insurance-based healthcare system designed to ensure equal access to healthcare services based on need, also encountered novel challenges during the pandemic. Korea’s robust control system for early detection and rapid national response was widely viewed as enabling the country to manage demand for both COVID-19 and non-COVID-19 healthcare services without full-scale lockdowns and strict movement restrictions [9]. However, Korea experienced a notable decrease in healthcare utilization during the early stages of the global pandemic in 2020 [10]. A survey conducted that year revealed that approximately 65% of Korean adults reported either avoiding or giving up on seeking healthcare services [11]. Furthermore, approximately 30% of respondents felt anxious due to their inability to access needed health services, with 13% ultimately reporting interruptions in care during the early phase of the pandemic [11]. Although supply-side constraints have been highlighted as major contributors to disrupted care during the pandemic, public perceptions may also have shaped healthcare-seeking decisions, underscoring the need to further investigate their relationship with delayed or foregone care.

Fears of virus transmission, distrust in the healthcare environment, or lack of confidence in safety protocols discouraged many individuals from seeking care unless it was an urgent health issue [12,13]. These behavioural responses appear to be shaped by how well the public understood and trusted the pandemic-related preventive measures [8,14]. Individuals who perceived a higher risk of infection or lacked confidence in public health protocols tended to avoid healthcare settings [15]. In contrast, those with greater confidence in safety protocols and trust in institutional responses were more likely to continue engaging with the healthcare system [15]. This highlights the importance of both analytical processing based on understanding and trust when making healthcare decisions under uncertainty [16]. This suggests that healthcare-seeking behaviour may be guided by understanding and trust functioning as parallel or compensatory mechanisms. By clarifying how public perceptions of the COVID-19 preventive measures shape individual experiences of delayed or foregone care, this study provides insights that may inform policy decisions on the extent to which governments and healthcare sectors need to engage in effective risk communication to ensure access to essential services [17]. Therefore, the aim of this study was to examine the association between public perceptions, particularly understanding and trust, and delayed or foregone care among young and middle-aged Korean adults.

## Materials and methods

This study used the 2020 Koreans’ Happiness Survey (KHS), a nationally representative cross-sectional survey, conducted by the National Assembly Future Institute (NAFI). The aim of the survey was to collect information on the overall happiness of Koreans for the development of a wide range of policy interventions for improving the happiness level of the population. The KHS was first introduced in 2020 and has been conducted annually. It collects information on a wide range of factors associated with happiness including social and psychological experiences and socio-demographic characteristics. The COVID-19 module data were only collected in the 2020 KHS in response to the global pandemic crisis. The module included information on an individual’s experience of healthcare services, their perceptions, beliefs, and experiences, and economic impacts of the global pandemic. The KHS surveyed 6,500 households and 14,300 people extracted from the general Korean population, using a multi-stage stratified cluster sampling method. The survey data were collected using Tablet-Assisted Personal Interviewing (TAPI). During each interview, the interviewer directly entered the respondent’s answers into a tablet in real time, which allowed immediate quality control and minimized non-response. The KHS was conducted using standard ethical procedures, and all participants provided written informed consent at the time of the original data collection. For this study, 11,307 survey respondents, between the ages of 19 and 64, who participated in both the general and COVID-19 module questionnaires, were included in the analyses. This study focused on working-age adults, who constitute the economically active population and are particularly susceptible to barriers to timely access due to competing demands from work, caregiving, and mobility during the pandemic. The ethics exemption was obtained from the Institutional Review Board of Seoul National University Hospital (IRB No. 2205-005-1320). The requirement for informed consent was waived by the IRB, as the study involved secondary analysis of a publicly available and fully anonymized dataset.

### Dependent variable – self-reported instances of delayed or foregone care

The dependent variable was the self-reported instance of delayed or foregone care after the COVID-19 outbreak in February 2020. To identify self-reported instances of delayed or foregone care, the following questions were utilized: – “*Did you feel the need to receive a medical examination or treatment at a hospital, clinic, or public health center for purposes other than diagnosis and treatment of COVID-19 after the outbreak of COVID-19 (since February 2020)*?” The possible responses were “*Yes*” or “*No*.” The respondents who responded they needed health services were then prompted to answer the question, *“Did you receive the needed care in a timely manner?*” There were three possible response options: “*Yes*,” “*No - received care late*,” and “*No - gave up*.” Self-reported instances of delayed or foregone care that occurred following the COVID-19 pandemic were classified accordingly based on responses to these two questions. Delayed or foregone care represents an inability to obtain timely or appropriate care when needed and is widely treated as a component of the same construct in existing health services research (11,29,30). In this study, delayed or foregone care is treated as a single composite outcome, encompassing both delayed receipt of care and complete forgoing of care when healthcare services were needed.

### Independent variable – Public perceptions of COVID-19 preventive measures

With respect to public perceptions of the COVID-19 preventive measures, the statements “*I know the COVID-19 preventive measures well*” and “*I trust the COVID-19 preventive measures*” were utilized to identify individual understanding and trust in the COVID-19 preventive measures, respectively. Both items were measured on five-point Likert scales ranging from 1 = strongly disagree to 5 = strongly agree and dichotomised into high (agree/strongly agree) versus low/neutral (all other responses) for analytical clarity and to ensure stable estimation. Based on these two indicators, a composite variable was constructed to classify public perceptions: (1) neither trust nor understanding, (2) trust only, (3) understanding only, and (4) both trust and understanding. This classification reflects the simultaneous presence or absence of perceived cognitive and affective perceptions of preventive measures, which are theorized to have influenced healthcare-seeking behaviour during the pandemic [18–20].

### Covariates

Previous studies and Andersen’s expanded health behavior model (HBM) were used to select factors that potentially influence the use of healthcare services [21,22]. According to the Andersen HBM, factors related to the use of healthcare services are classified into predisposing factors, enabling factors and need factors. Predisposing factors include demographic characteristics such as age groups (19−34, 35−49, 50−64), gender, and region (Seoul metro region vs. non-Seoul metro regions). Enabling factors include socioeconomic status such as income, educational attainment, and employment status. Need factors are physiological and psychological factors affecting the use of healthcare services such as chronic condition, self-rated health, changes in health since the pandemic onset, and COVID-19 risk perception.

### Statistical analyses

Prior to regression analyses, multicollinearity among variables included in the analytic models was assessed using variance inflation factors (VIF). All VIF values were below 2.5, suggesting that multicollinearity was not a concern. Logistic regression analysis was conducted after covariate adjustment to examine whether public perceptions of the COVID-19 preventive measures is associated with the experience of delayed or foregone care among Korean adults.

To assess the robustness, supplementary analyses included an alternative age specification (including respondents aged ≥65 years), re-estimation excluding the perception variable, and tests of interaction terms between perceptions and selected socioeconomic factors (age, sex, income, and education). Given the low event rate (4.0%) of delayed or foregone care, rare-events logistic regression was also conducted as an alternative specification [23].

Data analyses were performed using Stata version 18 (StataCorp LLC, College Station, TX, USA). The results are presented as odds ratios (OR) and 95% confidence intervals (95% CI). All analyses accounted for the complex sampling design of the Korea Health Survey by using STATA’s svy commands, which adjust for sampling weights, clustering, and stratification, in accordance with the official data documentation. The sampling weights incorporated adjustments for differential selection probability and non-response, allowing population-representative estimation.

## Results

Table 1 shows the general characteristics of the study population, stratified by delayed or foregone care. Among the 11,307 working-age adults aged 19–64 included in the analysis, 448 (4.0%) of all respondents experienced delayed or foregone care in 2020. This is considerably lower than pre-pandemic estimates, the 10–20% rate of difficulties in accessing or utilizing healthcare among Korean adults, possibly reflecting differences in question wording, reference period, and population coverage [24,25]. Individuals who reported an experience of delayed or foregone care were more likely to be younger, have poorer self-rated health, have worsened health since the pandemic, and reside in the Seoul metropolitan region. Delayed or foregone care was also more common among individuals with lower levels of education, lower income levels, and high perceived risk of COVID-19. Furthermore, delayed or foregone care was more frequently reported among those who expressed neither trust nor understanding of COVID-19 preventive measures.

**Table 1:** General characteristics of study subjects regarding delayed or foregone care– 2020 Koreans’ Happiness Survey (n = 11,307).

Variables		Delayed or foregone care		
		Yes – n/mean (n = 448)	No – n/mean (n = 10859)	p-value
Gender	Male	196	5119	0.12
	Female	252	5740	
Age	19-34	96	2681	0.03
	35-49	121	3331	
	50-64	231	4847	
Chronic condition	0	311	9926	<0.05
	1-2	73	301	
	≥3	64	632	
Self-rated health	Good	406	10533	<0.05
	Bad	42	326	
Change in health since the pandemic	Better	102	1814	<0.05
	Same	303	8655	
	Worse	43	390	
Education	Middle school or under	5	364	<0.05
	High school	173	3660	
	College or above	270	6835	
Income	Q1	101	2515	0.12
	Q2	78	1898	
	Q3	131	3035	
	Q4	104	2207	
	Q5	34	1204	
Economic activity	Employed	250	6069	0.06
	Self-employed	71	1389	
	Non-paid family business	11	206	
	Unemployed	116	3195	
Region	Seoul metro region	302	4619	<0.05
	Non-Seoul metro regions	146	6240	
Perception of COVID-19 preventive measures	Neither trust nor understanding	115	2137	<0.05
	Trust only	73	1732	
	Understanding only	92	1727	
	Both trust and understanding	168	5263	
COVID-19 risk perception	Low perceived risk	127	3585	<0.05
	High perceived risk	321	7274	

Note: Counts are unweighted. Statistical tests were conducted using survey weights to account for the complex sampling design.

Table 2 presents the results of the logistic regression analysis. Individuals who reported both trust and understanding were significantly less likely to experience delayed or foregone care (OR: 0.49, 95% CI: 0.36–0.65) as compared to individuals with neither trust nor understanding of the preventive measures. In contrast, there was no association with delayed or foregone care by those reporting either trust or understanding. Sensitivity analyses yielded results consistent with the main analysis, with no additional statistically significant associations observed (results not shown). Results from the rare-events logistic regression are presented in S1 Table.

**Table 2:** Association between public perceptions of COVID-19 preventive measures and delayed or foregone care– 2020 Koreans’ Happiness Survey (n = 11,307).

Variables	Delayed or foregone care	
	OR	95% CI
Perception of COVID-19 preventive measures		
(ref. Neither trust nor understanding)		
Trust only	0.88	0.63-1.23
Understanding only	0.78	0.54-1.14
Both trust and understanding	0.49	0.36-0.65
Gender (ref. Male)		
Female	1.09	0.84-1.42
Age (ref. 19–34)		
35-49	0.81	0.59-1.11
50-64	0.96	0.69-1.32
Chronic condition (ref. 0)		
1-2	5.85	4.18-8.20
≥3	3.31	2.32-4.74
Self-rated health (ref. Good)		
Bad	1.35	0.88-2.09
Change in health since the pandemic (ref. Better)		
Same	0.78	0.60-1.02
Worse	2.15	1.36-3.39
Education (ref. College or above)		
Middle school or under	0.17	0.06-0.47
High school	0.83	0.63-1.08
Income (ref. Q5)		
Q1	2.89	1.50-5.54
Q2	2.44	1.43-4.17
Q3	2.17	1.37-3.46
Q4	1.99	1.27-3.14
Economic activity (ref. Employed)		
Self-employed	1.18	0.85-1.63
Non-paid family business	0.99	0.46-2.11
Unemployed	0.59	0.36-0.96
Region (ref. Seoul metro region)		
Non-Seoul metro regions	0.29	0.23-0.37
COVID-19 risk perception (ref. Low perceived risk)		
High perceived risk	1.57	1.22-2.01

Aside from the COVID-19 measures, chronic condition was associated with a higher likelihood of delayed or foregone care. Compared to individuals without any chronic condition, those with 1–2 chronic conditions (OR: 5.85, 95% CI: 4.18–8.20) or 3 or more conditions had higher odds (OR: 3.31, 95% CI: 2.32–4.74) of delayed or foregone care. Deterioration in health status since the onset of the pandemic was also associated with delayed or foregone care. Respondents who reported worsened health had significantly greater odds of experiencing care delay (OR: 2.15, 95% CI: 1.36–3.39), compared to those whose health had improved. With respect to socioeconomic factors, income level showed a clear inverse gradient. The odds of delayed or foregone care were significantly higher for those in the lowest quintile (OR: 2.89, 95% CI: 1.50–5.54) as compared to those in the highest income quintile. Elevated odds were also observed in the second (OR: 2.44, 95% CI: 1.43–4.17), third (OR: 2.17, 95% CI: 1.37–3.46), and fourth quintiles (OR: 1.99, 95% CI: 1.27–3.14). Individuals with middle school or less education were significantly less likely to report delayed or foregone care (OR: 0.17, 95% CI: 0.06–0.47) compared to those with a college education or higher. Regional variation was also evident. Respondents living in non-Seoul metropolitan regions had significantly lower odds of delayed or foregone care (OR: 0.29, 95% CI: 0.23–0.37) compared to those in the Seoul metropolitan region. Finally, individuals with high perceived risk of COVID-19 had significantly higher odds of experiencing delayed or foregone care (OR: 1.57, 95% CI: 1.22–2.01).

## Discussion

This study aimed to examine an association between public perceptions of COVID-19 preventive measures, with particular emphasis on understanding and trust, and instances of delayed or foregone care amid the global pandemic among young and middle-aged Korean adults. Utilizing the 2020 KHS, a nationally collected survey dataset, analyses showed that individuals with trust and understanding of the COVID-19 preventive measures were less likely to have instances of delayed or foregone care. Apart from public perceptions of the preventive measures, those with lower incomes tended to report more instances of delayed or foregone care. Meanwhile, individuals residing in non-Seoul metro regions and with the lowest levels of education tended to have lower odds of delayed or forgone care during the early stages of the global pandemic.

Healthcare-seeking behaviours are often related to perceived risk of highly contagious disease transmission, such as COVID-19, making public compliance with health authorities’ recommendations a central feature of risk management [26,27]. During COVID-19, even where preventive measures were scientifically reliable, many individuals nevertheless encountered instances of delayed or foregone care [28–33]. The findings from this study highlight the importance of both trust and understanding in these measures as major factors in healthcare use under crisis conditions. Trust, a subjective belief that reduces uncertainty and perceived risk when knowledge is incomplete, might contribute to a sense of safety and may encourage healthcare seeking [34]. In the pandemic context, such trust was closely linked to prevailing public opinion, government communication, and media narratives [19,35,36]. Conversely, lack of trust in the preventive measures may amplify fears and uncertainty, increase perceived barriers to healthcare services, ultimately contributing to delayed or foregone care [33]. Similarly, a clear understanding of preventive measures – reflecting an individual’s level of knowledge – enhances confidence in safely accessing care, thereby reducing fear and hesitation [37,38]. From a health policy perspective, it is therefore essential to ensure that the public is well informed with science-based evidence to promote behavioural compliance and prevent disruptions to necessary services during crises. Risk communication strategies should prioritise both clarity and credibility to mitigate fear-driven avoidance of healthcare services [39].

Aside from the public perceptions of preventive measures, individuals who reported a deterioration in their health following the global pandemic and those with chronic conditions had a higher likelihood of reporting delayed or foregone care. This pattern may suggest that individuals with greater healthcare needs may have faced disproportionate barriers to timely access during the pandemic. There are some plausible explanations for this association. Disruptions in routine services, postponed diagnoses, and gaps in chronic disease management may have been particularly consequential for those requiring continuous care, potentially reinforcing cycles of health deterioration [40–42]. In addition, elevated vulnerability to infection among individuals with chronic conditions may have increased avoidance of healthcare settings during periods of uncertainty. Taken together, these findings highlight the importance of maintaining continuity of essential services for populations with higher healthcare needs during public health crises.

In addition to the primary findings, this study reaffirms the important role of socioeconomic status in delayed or foregone care, even under a universal healthcare system. Demonstrating the persistence of financial barriers to healthcare services, the finding aligns with previous analyses [25,43,44], suggesting that Korea’s healthcare system, although designed to minimize financial obstacles, may fall short in practice. This persistent income gradient may reflect hypothesised mechanisms in existing literature including liquidity constraints, heightened cost sensitivity, and increased risk aversion during uncertain conditions [25,43,44]. These mechanisms may have been particularly salient during the early phase of the pandemic, when uncertainty about work and household income was high. Under such circumstances, individuals with limited financial reserves may have been more reluctant to incur healthcare expenses or take time away from work to receive care, increasing the likelihood of delaying or foregoing care. These mechanisms could help explain why income remains a robust predictor of delayed or foregone care, even under universal coverage. By contrast, the education results moved in the opposite pattern. This finding contrasts with existing evidence [25,29,45] and may be attributed to behavioural or perceptual differences. Individuals with lower education may have lower expectations of healthcare, limited awareness of available services, or lower health literacy, all of which can reduce the likelihood of reporting delayed or foregone care [45,46]. Alternatively, long-standing disengagement from the healthcare system may also make care disruptions less visible among those with the least education. This counterintuitive pattern may also reflect unmeasured confounding with employment security or occupational type, as well as reporting bias, whereby individuals with lower education may be less likely to identify or report disruptions in access to care even when they occur. Taken together, these results highlight the call for policy strategies that minimize socioeconomic barriers, particularly related to income and education, in order to improve healthcare accessibility in preparation for future public health crises.

This study analyzed nationally representative cross-sectional data from the KHS COVID-19 module, collected at a single time point in 2020, to examine the association between public perceptions of preventive measures and delayed or foregone care. Several limitations need to be acknowledged. First, the cross-sectional design precludes causal inference. Temporal ordering cannot be confirmed, and the findings may be subject to reverse causality, meaning that experiences of delayed or foregone care may have shaped individuals’ trust or understanding of COVID-19 preventive measures rather than the reverse. Endogeneity concerns also remain, including omitted variable bias, as unmeasured psychological traits, health beliefs, or prior healthcare experiences may influence both public perceptions and healthcare-seeking behaviours. Second, the study relied on self-reported outcomes, which are subject to recall error or social desirability bias. These are inherent limitations of secondary survey-based research and warrant cautious interpretation of the results. Third, the timing of data collection—during the early phase of the pandemic—limits the generalisability of the findings to subsequent phases of the public health responses. Unlike countries that conducted monthly or wave-specific tracking of healthcare access or utilisation [47,48], the KHS collected COVID-19 module data at a single point in time. Consequently, changes in healthcare-seeking behaviour across different phases of the pandemic when infection levels, vaccination, and preventive could not be captured. Moreover, variation in the spread of COVID-19 and its consequences for healthcare utilisation varied across regions and population subgroups in Korea, which could not be fully addressed. Fourth, although the use of secondary data allowed timely and nationally representative analysis, the survey did not include detailed information on reasons for delaying or foregoing care—such as fear of infection, logistical constraints, or financial hardship. The absence of these details restricted the ability to identify specific behavioural mechanisms and to distinguish supply-side constraints such as clinic closures, or restricted hours from demand-side factors such as fear of infections and reduced perceived need for health services. Future surveys should incorporate such items to enable more precise analysis. Finally, trust and understanding of COVID-19 preventive measures were each assessed using single binary indicators in this study. This oversimplified complex perceptions and did not capture gradations in trust or knowledge. Although analytically practical, this binary categorization reduces the granularity of perceptions and may not fully capture variation in the intensity of trust or knowledge, potentially introducing measurement error and attenuating the magnitude of the observed association. Future studies using multi-item scales or latent modelling approaches may provide a more nuanced assessment of cognitive and affective dimensions of perceptions.

## Supporting information

S1 Table Results of rare event logistic regression analysis. (DOCX)
