# Supplementary material for: Navigating COVID-19: Association between public perceptions of preventive measures and delayed or foregone care among young and middle-aged Korean adults in the early phase of the pandemic
Source: PLoS One. 2026 Mar 13;21(3):e0344209. doi: 10.1371/journal.pone.0344209 (PMC12987425; doi:10.1371/journal.pone.0344209)
Supplement: S1 Table — (DOCX) [file pone.0344209.s001.docx]

**Supplementary**

S1 Table. Results of rare event logistic regression analysis

| Variables | Delayed or foregone care | |
| --- | --- | --- |
|  | OR | 95% CI |
| Perceptions of COVID-19 preventive measures |  |  |
| (ref. Neither trust nor understanding) |  |  |
| Trust only | 0.88 | 0.63-1.23 |
| Understanding only | 0.79 | 0.54-1.14 |
| Both trust and understanding | 0.49 | 0.37-0.65 |
| Gender (ref. Male) |  |  |
| Female | 1.09 | 0.84-1.42 |
| Age (ref. 19-34) |  |  |
| 35-49 | 0.81 | 0.59-1.11 |
| 50-64 | 0.96 | 0.69-1.32 |
| Chronic condition (ref. 0) |  |  |
| 1-2 | 5.81 | 4.15-8.14 |
| ≥3 | 3.31 | 2.32-4.73 |
| Self-rated health (ref. Good) |  |  |
| Bad | 1.36 | 0.88-2.09 |
| Change in health since the pandemic (ref. Better) | |  |
| Same | 0.78 | 0.60-1.02 |
| Worse | 2.15 | 1.36-3.38 |
| Education (ref. College or above) |  |  |
| Middle school or under | 0.19 | 0.07-0.53 |
| High school | 0.83 | 0.63-1.08 |
| Income (ref. Q5) |  |  |
| Q1 | 2.79 | 1.36-3.38 |
| Q2 | 2.42 | 1.42-4.12 |
| Q3 | 2.15 | 1.35-3.41 |
| Q4 | 1.97 | 1.27-3.11 |
| Economic activity (ref. Employed) |  |  |
| Self-employed | 1.18 | 0.86-1.63 |
| Non-paid family business | 1.04 | 0.49-2.23 |
| Unemployed | 0.60 | 0.37-0.98 |
| Region (ref. Seoul metro region) |  |  |
| Non-Seoul metro regions | 0.30 | 0.23-0.38 |
| COVID-19 risk perception (ref. Low perceived risk) |  |  |
| High perceived risk | 1.56 | 1.22-2.01 |

Note: Rare-events logistic regression (King & Zeng, 2001) was used to correct small-sample bias in logistic estimation given the low event rate (4%) of delayed or foregone care in the 2020 Koreans’ Happiness Survey.
